# Respiratory pulse pressure variation fails to predict fluid responsiveness in acute respiratory distress syndrome

**DOI:** 10.1186/cc10083

**Published:** 2011-03-07

**Authors:** Karim Lakhal, Stephan Ehrmann, Dalila Benzekri-Lefèvre, Isabelle Runge, Annick Legras, Pierre-François Dequin, Emmanuelle Mercier, Michel Wolff, Bernard Régnier, Thierry Boulain

**Affiliations:** 1 Service de réanimation médicale et maladies infectieuses, Hôpital Bichat-Claude Bernard, Assistance Publique des Hôpitaux de Paris, 18 rue Henri Huchard, F-75018 Paris, France; 2 Service de réanimation médicale polyvalente, centre hospitalier régional universitaire de Tours, 2 boulevard Tonnelé, F-37044 Tours, France; 3 Service de réanimation médicale, Hôpital La Source, centre hospitalier régional, avenue de l'Hôpital, F-45067 Orléans cedex 1, France

## Abstract

**Introduction:**

Fluid responsiveness prediction is of utmost interest during acute respiratory distress syndrome (ARDS), but the performance of respiratory pulse pressure variation (Δ_RESP_PP) has scarcely been reported. In patients with ARDS, the pathophysiology of Δ_RESP_PP may differ from that of healthy lungs because of low tidal volume (Vt), high respiratory rate, decreased lung and sometimes chest wall compliance, which increase alveolar and/or pleural pressure. We aimed to assess Δ_RESP_PP in a large ARDS population.

**Methods:**

Our study population of nonarrhythmic ARDS patients without inspiratory effort were considered responders if their cardiac output increased by >10% after 500-ml volume expansion.

**Results:**

Among the 65 included patients (26 responders), the area under the receiver-operating curve (AUC) for Δ_RESP_PP was 0.75 (95% confidence interval (CI_95_): 0.62 to 0.85), and a best cutoff of 5% yielded positive and negative likelihood ratios of 4.8 (CI_95_: 3.6 to 6.2) and 0.32 (CI_95_: 0.1 to 0.8), respectively. Adjusting Δ_RESP_PP for Vt, airway driving pressure or respiratory variations in pulmonary artery occlusion pressure (ΔPAOP), a surrogate for pleural pressure variations, in 33 Swan-Ganz catheter carriers did not markedly improve its predictive performance. In patients with ΔPAOP above its median value (4 mmHg), AUC for Δ_RESP_PP was 1 (CI_95_: 0.73 to 1) as compared with 0.79 (CI_95_: 0.52 to 0.94) otherwise (*P *= 0.07). A 300-ml volume expansion induced a ≥2 mmHg increase of central venous pressure, suggesting a change in cardiac preload, in 40 patients, but none of the 28 of 40 nonresponders responded to an additional 200-ml volume expansion.

**Conclusions:**

During protective mechanical ventilation for early ARDS, partly because of insufficient changes in pleural pressure, Δ_RESP_PP performance was poor. Careful fluid challenges may be a safe alternative.

## Introduction

Many appealing indices have been proposed to predict fluid responsiveness, using heart-lung interactions (for example, respiratory variations of pulse pressure (Δ_RESP_PP)) [1,2] or passive leg raising [3]. Δ_RESP_PP requires controlled mechanical ventilation in nonarrhythmic patients sufficiently sedated for not triggering the ventilator [4]. As the use of sedation in the intensive care unit (ICU) has decreased over the past few years, this situation is rarely encountered, except in cases such as severe respiratory failure (such as acute respiratory distress syndrome (ARDS)) requiring perfect patient-ventilator interactions. Of note, fluid responsiveness prediction is crucial in patients with ARDS because of increased alveolar-capillary membrane permeability [5], and avoiding unnecessary fluid loading has been shown to have a positive effect on patient outcome [6].

Nevertheless, cardiopulmonary interactions are complex in case of ARDS, particularly when lung-protective mechanical ventilation (low tidal volume) is performed as recommended nowadays [5], and several limitations may downplay the usefulness of Δ_RESP_PP. First, the magnitude of the insufflated tidal volume (Vt) affects the magnitude of Δ_RESP_PP (or other indices derived from respiratory changes in stroke volume) in non-ARDS or mixed ARDS and non-ARDS patients [7-9]. Thus, the performance of Δ_RESP_PP becomes poor when the Vt is settled below 8 ml/kg [10,11]. Second, ARDS patients exhibit a marked decrease in lung and sometimes chest wall compliance [5]. Consequently, airway driving pressure (plateau pressure (Pplat) minus total positive end-expiratory pressure (PEEPt)) for a given Vt is greater in ARDS than in healthy lungs [12]. Therefore, it has been hypothesized that, despite a reduced Vt, cyclic swings in airway pressure are still high enough to maintain Δ_RESP_PP predictive ability in ARDS patients [13]. However, one may question this assumption. Indeed, Δ_RESP_PP results of swings in right atrial pressure which are close to pericardial and pleural pressure swings. Rather than airway driving pressure, the main determinants of respiratory changes in pleural, pericardial and atrial pressure are Vt magnitude and chest wall compliance (both of which determine the compression of the anatomic structures in the cardiac fossa) [14,15]. Decreased lung compliance during ARDS may therefore have little effect on Δ_RESP_PP [12]. Last, to avoid respiratory acidosis, reduced Vt is frequently combined with an increased respiratory rate (RR), which may also downplay the performance of Δ_RESP_PP [16].

Thus, Δ_RESP_PP may be of interest to guide fluid therapy during ARDS, but several physiological mechanisms may limit its validity. The current literature about its performance in ARDS is scarce, and opposite conclusions have been drawn [10,17]. We aimed to assess the performance of Δ_RESP_PP to predict fluid responsiveness in a large population of patients with ARDS.

## Materials and methods

ARDS patients from another study were studied [3] and are being partly shared with another study [18]. In the three participating centers (Hôpital Bichat-Claude Bernard, Paris, France; Centre Hospitalier Régional Universitaire of Tours, Tours, France; and Centre Hospitalier Régional of Orléans, Orléans, France), patients were included over the same 18-month period, either after written informed consent was obtained from a relative or after emergency enrollment followed by delayed consent as approved by our regional ethics board.

### Patients

Adults with acute circulatory failure (systolic blood pressure <90 mmHg, mean blood pressure <65 mmHg, skin mottling, urine output <0.5 ml/kg/hour, arterial lactate >2.5 mM/l or vasopressor infusion) and ARDS [19] exhibiting a Ramsay sedation scale score >4 and no arrhythmia were included if they were receiving mechanical ventilation in volume-controlled mode without triggering the ventilator.

Patients were not included if they were receiving diuretic treatment, had uncontrolled hemorrhage, were in a state of brain death, were receiving intraaortic balloon pump support, had a risk of fluid loading-induced, life-threatening, hypoxemia (partial pressure of O_2 _to fraction of inspired O_2 _ratio (PaO_2_/FiO_2 _ratio) <70 mmHg, body weight indexed extravascular lung water (EVLWi) >22 ml^-1 ^kg^-1 ^(PiCCO™ system: Pulsion Medical Systems AG, Munich, Germany), transmural pulmonary artery occlusion pressure (PAOPtm) >22 mmHg (pulmonary artery catheter; Edwards Lifesciences, Irvine, CA, USA)). PAOPtm equals PAOP minus an estimation of the extramural pressure that acts on pulmonary vessels and was calculated as follows: PAOPtm = end expiratory PAOP - [PEEPt × (end inspiratory PAOP - end expiratory PAOP)/(Pplat - PEEPt)]) [20].

The study procedure was stopped in case of changes in respirator settings or vasoactive therapy, occurrence of arrhythmia or respiratory intolerance to volume expansion (EVLWi >22 ml^-1 ^kg^-1 ^or PAOPtm >22 mmHg or 5% decrease in pulse oxymetry (SpO_2_)). Mechanical ventilation, vasoactive therapy, sedation and paralysis were set by the attending physician and not modified.

### Measurements

Hemodynamic (heart rate (HR), blood pressure and cardiac output (CO)) and respiratory parameters (PEEPt, Pplat, RR and Vt) were measured at baseline, immediately after infusion of 300 ml of modified fluid gelatin over 18 minutes (to assess the respiratory tolerance) and an additional 200 ml over 12 minutes.

CO was measured through end-expiratory injection of 10 ml or 15 ml (transcardiac or transpulmonary thermodilution, respectively) of an iced dextrose solution (using a closed injection system with in-line temperature measurement: CO-set+™ system (Edwards Lifesciences) or that which is included in the PiCCO™ system). Three consecutive measurements within 10% (if not, seven measurements) were averaged.

The correct placement of the pulmonary artery catheter was ascertained by visualization of concordant waveforms and calculation of the respiratory changes in PAOP (ΔPAOP)-to-respiratory changes in pulmonary artery pressure (ΔPAP) ratio [21].

Central venous pressure (CVP) (direct reading of the displayed value), PAOP (end-expiratory value measured on frozen waveform) and blood pressure were measured with a disposable transducer (TruWave™; Baxter Division Edwards, Maurepas, France), zeroed at the level of the midaxillary line. Offline, on high-resolution paper tracings, including airway and blood pressure waveforms and after their numerical enlargement, Δ_RESP_PP was calculated by an observer blinded to other hemodynamic data as follows and averaged over three consecutive respiratory cycles:

within one respiratory cycle [1]. Other indices derived from respiratory changes in arterial pressure were calculated over three consecutive respiratory cycles: the expiratory decrease in systolic pressure (dDown) and the respiratory changes in systolic pressure (SPV) [15].

Echocardiography was performed within 6 hours of measurements to quantify valvular regurgitations and to detect intracardiac shunts or acute *cor pulmonale *(right-to-left ventricular end-diastolic area ratio above 0.6 with paradoxical septal wall motion).

### Statistical analysis

Patients were classified as responders if volume expansion induced an increase in CO ≥10% and as nonresponders otherwise. Indeed, a measured increase of CO above 9% (which we rounded to 10%) reliably reflects that a real change has taken place [22]. To validate this choice of cutoff in our patients (assessment of intermeasurement variability within each set of measurements), we calculated the least significant change (LSC) for each set of CO measurements in each patient at each phase ((1.96√2)CV/√number of measurements within one set) with CV being the coefficient of variation (SD/mean). Thus, we ascertained that each individual patient classified as a responder had a CO increase above LSC [23]. Calculations were also performed using a 15% relative [1,4] or an absolute 300 ml/min/m^2 ^[24] cutoff to define fluid responsiveness.

Variables (expressed as means ± SD or *n *(%)) were compared using Student's *t*-test and Fisher's exact test (between responders and nonresponders), paired Student's *t*-test (for each patient), analysis of variance and the χ^2 ^test (between centers). For each index (Δ_RESP_PP, SPV and dDown), we calculated the area under the receiver-operating characteristic curve (AUC), determined positive and negative likelihood ratios (LR+ and LR-) for the best cutoff (Youden method) and for the widely used cutoff of 12% for Δ_RESP_PP [2]. The values of 5 and 10 for LR+ (or 0.2 and 0.1 for LR-) helped to divide the continuous scale of likelihood ratios into three categories: weak, good and strong evidence of discriminative power [25]. AUC values in subgroups of patients were compared [26]. *P *< 0.05 was considered statistically significant. All statistical tests were two-tailed and performed using MedCalc software (Mariakerke, Belgium) and Statview software (SAS Institute, Cary, NC, USA).

## Results

Sixty-five patients were included (Table 1). The mean LSCs of CO measurements were 6.7% and 6.5% at baseline and after volume expansion, respectively, and all responders exhibited individual CO changes from baseline to after volume expansion greater than their individual LSCs. Administration of catecholamine was the sole criterion triggering inclusion in 14 patients (22%): norepinephrine (*n *= 13, 0.40 ± 0.46 μg/kg/min) or epinephrine (*n *= 1, 0.26 μg/kg/min). Volume expansion was interrupted in two patients after 300-ml intolerance (one because of a 6% drop in SpO_2 _and one because of an increased EVLWi >22 ml/kg). Data after 300-ml volume expansion were used for analysis of these two patients. Hemodynamic parameters at baseline and their evolution after volume expansion are detailed in Table 2. The proportion of responders, the Simplified Acute Physiology Score II, baseline mean arterial pressure, HR, CO, and Δ_RESP_PP were similar between centers (all *P *> 0.05).

**Table 1:** Main characteristics of the patients at the time of inclusion^a^

Patient characteristic	Data
Age, yr	59 ± 15
Sex, male/female	45/20
SAPS II score	56 ± 19
Main diagnosis at admission, *n*	
Septic shock	28
Acute respiratory failure	12
Other	25
Delay between admission and study inclusion, *n *(%)	
<24 hours	42 (65%)
24 to 48 hours	12 (18%)
>48 hours	11 (17%)
Ramsay score 5 versus 6, *n*	14 versus 51
Responders using 10% versus 15% CO change to define fluid responsiveness, *n *(%)	26 (40%) versus 21 (32%)
Arterial lactate concentration, mM/l (*n *= 61)	3.0 ± 2.5
Arterial lactate concentration >2.5 mM/l, n (%)	25 (38%)
Urine output during the past hour, ml/kg	0.8 ± 0.8
Urine output during the last hour <0.5 ml/kg, n (%)	22 (34%)
Skin mottling, n (%)	22 (34%)
Catecholamine infusion, n (%)	59 (91%)
Norepinephrine, μg/kg/min (*n *= 53)	0.76 ± 0.88
Epinephrine, μg/kg/min (*n *= 10)	0.59 ± 0.49
Dobutamine, μg/kg/min (*n *= 20)	13 ± 10
CO measured by PiCCO™/versus pulmonary artery catheter, n (%)	32 (49%)/33 (51%)
Arterial catheter site, femoral versus radial, n (%)	51 (78%)/14 (22%)
PEEPt, cmH_2_O	8.5 ± 3.2
Plateau pressure, cmH_2_O	21.2 ± 5.0
Driving pressure (plateau pressure - PEEPt cmH_2_O)	13.7 ± 4.1
Alveolar to vascular pressure transmission index (*n *= 33) [20]	0.39 ± 0.17
Respiratory changes in PAOP, mmHg (*n *= 33)	4.8 ± 2.0 (range, 2 to 9)
Tidal volume, ml	457 ± 67
Tidal volume indexed to measured versus predicted body weight, ml/kg	6.5 ± 1.4 versus 6.9 ± 0.95
Respiratory system static compliance, ml/cmH_2_O	40.4 ± 15.8
RR, cycles/minute	24 ± 6
HR:RR ratio	4.5 ± 1.6
I:E ratio, %	31 ± 6
PaO_2_:FiO_2 _ratio, mmHg	136 ± 50

^a^SAPS, simplified acute physiology score II; CO, cardiac output; PEEPt; total positive end-expiratory pressure; PAOP, pulmonary artery occlusion pressure; I:E, inspiration length:expiration length ratio. HR:RR, heart rate:respiratory rate ratio.

Quantitative variables are expressed as mean ± SD.

**Table 2:** Hemodynamic parameters at baseline and after 500 ml volume expansion^a^

	Before volume expansion		After volume expansion	
		
Hemodynamic parameter	Responders	Nonresponders	Responders	Nonresponders
Heart rate, beats/min	101 ± 25	99 ± 24	98 ± 25^c^	95 ± 23^c^
Arterial pressure, mmHg	68 ± 12	73 ± 12	80 ± 16^c^	80 ± 14^c^
Central venous pressure, mmHg	9.5 ± 4.3	11.8 ± 4.4^b^	12.3 ± 4.8^c^	15.6 ± 4.8^c^
PAOP, mmHg (*n *= 33)	9.6 ± 3.3	13.2 ± 3.7^b^	14.9 ± 6.1^c^	17.5 ± 3.7^c^
Transmural PAOP (*n *= 33) [20]	6.2 ± 3.8	10.1 ± 3.9^b^	10.9 ± 6.5^c^	14.2 ± 4.1^c^
pulse pressure (mmHg)	49 ± 14	56 ± 14^b^	64 ± 18^c^	59 ± 16
Δ_RESP_PP, %	7.4 ± 5.2	3.8 ± 4.2^b^	4.9 ± 4.2^c^	2.9 ± 3
dDown, mmHg (*n *= 45)	6.5 ± 4.4	1.8 ± 2.5^b^	1.9 ± 5.4^c^	1.2 ± 1.6
SPV, mmHg	5.7 ± 4.3	2.8 ± 2.8^b^	4.8 ± 3.2^c^	2.2 ± 1.6
Pulmonary arterial pressure, mmHg (*n *= 33)	25 ± 6	29 ± 5^b^	29 ± 7^c^	35 ± 6 ^c^
Cardiac index, l/min/m^2^	3.3 ± 1.5	3.6 ± 1.4	4.2 ± 1.8^c^	3.5 ± 1.4

^a^PAOP, pulmonary artery occlusion pressure; Δ_RESP_PP, respiratory variations of pulse pressure; dDown, difference between the average, over three consecutive respiratory cycles, of the minimal value of systolic blood pressure during a respiratory cycle and the value of systolic blood pressure during apnea; SPV, respiratory changes in systolic arterial pressure over three consecutive respiratory cycles; ^b^*P *< 0.05 (responders versus nonresponders); ^c^*P *< 0.05 for comparison between before and after volume expansion.

Quantitative variables are expressed as mean ± SD.

### Predictive performance

**Figure 1:**
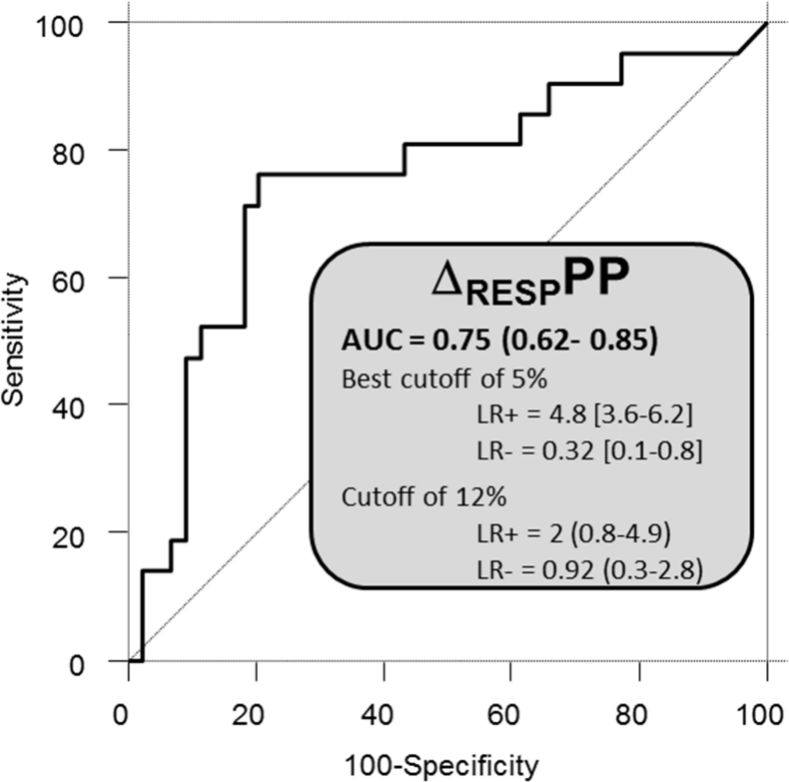
**Performance of respiratory changes in pulse pressure (Δ_RESP_PP) in the whole shocked acute respiratory distress syndrome (ARDS) population (*n *= 65)**. Receiver-operating characteristic (ROC) curve obtained for Δ_RESP_PP to predict a 10% increase in cardiac output after 500 ml volume expansion. AUC, area under the ROC curve. LR+, positive likelihood ratio. LR-, negative likelihood ratio.

**Figure 2:**
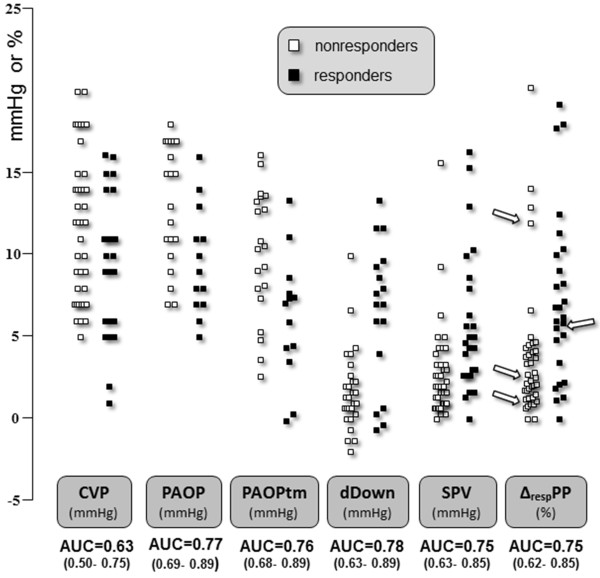
**Individual values of baseline static and breath-derived indices in responders and nonresponders**. CVP, central venous pressure; PAOP; pulmonary artery occlusion pressure; PAOPtm, transmural pulmonary artery occlusion pressure (see Materials and methods section for details) [20]; Δ_RESP_PP, respiratory changes in arterial pulse pressure; dDown, expiratory decrease in systolic arterial pressure; SPV, respiratory changes in systolic arterial pressure; AUC, area under the receiver-operating characteristic curve. Responders are defined as patients increasing their cardiac output by at least 10% after a 500-ml volume expansion. The arrows show patients with acute *cor pulmonale *(see Materials and methods section for definition).

Δ_RESP_PP was associated with an AUC of 0.75 (95% confidence interval (CI_95_): 0.62 to 0.85) and a best cutoff value of 5% (LR+ and LR- of 4.8 (CI_95_: 3.6 to 6.2) and 0.32 (CI_95_: 0.1 to 0.8), respectively) (Table 3 and Figures 1 and 2). The common 12% cutoff [2,17] was associated with LR+ and LR- values of 2 (CI_95_: 0.8 to 4.9) and 0.92 (CI_95_: 0.3 to 2.8), respectively.

**Table 3:** Predictive performance of Δ_RESP_PP according to chosen cutoff and fluid responsiveness definition^a^

Definition of fluid responsiveness	Increase in CO >10% after volume expansion		Increase in CO >15% after volume expansion		Increase in CO >300 ml/min/m^2 ^after volume expansion	
			
AUC for Δ_RESP_PP	0.75 (0.62 to 0.85)		0.75 (0.63 to 0.85)		0.76 (0.63 to 0.84)	
			
Cutoff for Δ_RESP_PP	12%	5%^b^	12%	5%^b^	12%	4%^b^
LR+	2 (0.8 to 4.9)	4.8 (3.6 to 6.2)	2.8 (1.2 to 6.8)	3.7 (2.8 to 4.9)	4.5 (2.2 to 9.5)	3.5 (2.6 to 4.7)
LR-	0.92 (0.3 to 2.8)	0.32 (0.1 to 0.8)	0.87 (0.3 to 2.6)	0.30 (0.1 to 0.8)	0.87 (0.1 to 6.0)	0.46 (0.2 to 1.1)
Se	0.15 (0.05 to 0.35)	0.73 (0.52 to 0.88)	0.19 (0.06 to 0.42)	0.76 (0.53 to 0.92)	0.16 (0.06 to 0.32)	0.62 (0.45 to 0.78)
Sp	0.92 (0.79 to 0.98)	0.85 (0.70 to 0.94)	0.93 (0.81 to 0.99)	0.80 (0.65 to 0.90)	0.96 (0.82 to 0.99)	0.82 (0.63 to 0.94)
PPV	0.57 (0.20 to 0.88)	0.76 (0.54 to 0.90)	0.57 (0.20 to 0.88)	0.64 (0.43 to 0.81)	0.86 (0.42 to 0.98)	0.82 (0.63 to 0.94)
NPV	0.62 (0.48 to 0.74)	0.83 (0.67 to 0.92)	0.71 (0.57 to 0.82)	0.88 (0.72 to 0.95)	0.47 (0.33 to 0.60)	0.62 (0.45 to 0.7)

^a^CO, cardiac output; AUC, area under the receiver operating characteristic curve; Δ_RESP_PP, respiratory changes in pulse pressure; LR+, positive likelihood ratio; LR-, negative likelihood ratio; Se, sensitivity; Sp, specificity; PPV; positive predictive value; NPV, negative predictive value; ^b^best cutoff identified in our study population. Ranges in parentheses represent 95% confidence intervals.

**Figure 3:**
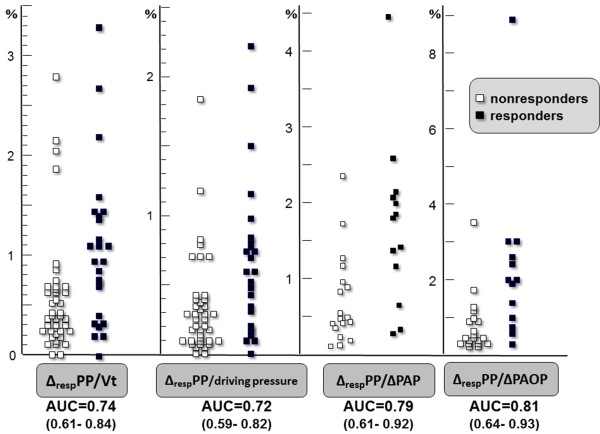
**Individual values of baseline respiratory changes in arterial pulse pressure (Δ_RESP_PP) corrected for surrogates of respiratory variations in pleural pressure**. Vt, tidal volume; driving pressure, airway plateau pressure minus total end-expiratory pressure; ΔPAOP: respiratory changes in pulmonary artery occlusion pressure; ΔPAP, respiratory changes in pulmonary artery pressure; AUC, area under the receiver-operating characteristic curve. Responders are defined as patients increasing their cardiac output of at least 10% after 500-ml volume expansion.

Adjusting Δ_RESP_PP for various estimates of extramural vascular pressure variations (Δ_RESP_PP/Pplat, Δ_RESP_PP/driving pressure, and Δ_RESP_PP/Vt ratios) did not lead to major improvement in predictive performance (Figure 3). In the 33 carriers of a pulmonary artery catheter, Δ_RESP_PP/ΔPAP and Δ_RESP_PP/ΔPAOP were associated with AUCs of 0.79 (CI_95_: 0.61 to 0.92) and 0.81 (CI_95_: 0.64 to 0.93), respectively. Figures 2 and 3 show the important overlap of baseline values of each index between responders and nonresponders.

**Figure 4:**
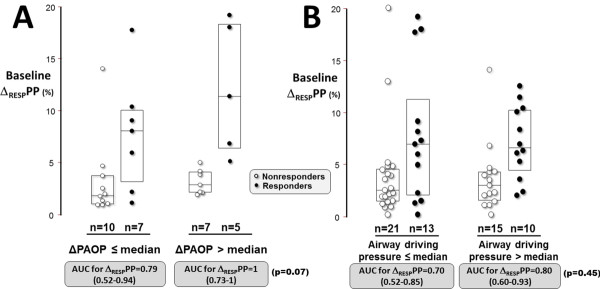
**Individual values of baseline Δ_RESP_PP according to volume responsiveness status and to either respiratory change in PAOP (ΔPAOP) or airway driving pressure**. For the purpose of this physiological analysis, patients with ultrasonographic signs of acute *cor pulmonale *were excluded. The central boxes represent the values from the lower to the upper quartile (25th to 75th percentile). The middle line represents the median. Δ_RESP_PP, respiratory changes in pulse pressure to predict a 10% increase in cardiac output after 500-ml volume expansion; AUC, area under the receiver-operating characteristic curve. **(A) **Analysis of the 33 patients with a pulmonary artery catheter. Median for respiratory changes in pulmonary artery occlusion pressure (PAOP) was 4 mmHg. Respiratory change in PAOP equals tidal volume (Vt) divided by chest wall compliance (see Additional file 1 for detailed calculations). Therefore, patients represented in the right part of the figure are those combining a higher Vt and lower chest wall compliance. **(B) **The median airway driving pressure was 10 cmH_2_O (*n *= 59).

With the purpose of identifying a subpopulation in which Δ_RESP_PP might achieve better results, we performed a subgroup analysis. In case of respiratory variation in PAOP above its median value (>4 mmHg), Δ_RESP_PP was associated with an AUC of 1 (CI_95_: 0.73 to 1) as compared with 0.79 (CI_95_: 0.52 to 0.94) otherwise (*P *= 0.07), with a marked decrease of the visual overlap of baseline values of Δ_RESP_PP between responders and nonresponders (Figure 4A). Dividing our whole population according to the median value of airway driving pressure (10 cmH_2_O) did not lead to marked difference in AUC and/or in the visual overlap (Figure 4B).

Overall, Δ_RESP_PP performed similarly in the subgroups of patients according to respiratory system compliance, norepinephrine dosage, administration of neuromuscular blocking agents (*n *= 26), site of the arterial catheter (radial (*n *= 14) or femoral (*n *= 51)) (Additional file 1). SPV (*n *= 65), dDown (*n *= 45), CVP (*n *= 65), PAOP (*n *= 33) and PAOPtm (*n *= 33) were associated with an AUC below 0.78 (Figure 2). All the results were similar when using a 15% relative or a 300 ml/min/m^2 ^absolute cutoff for volume expansion-induced increase in CO to define fluid responsiveness (Table 3 and Additional file 1, Figures S1 and S2). Among the 40 patients whose CVP increased by ≥2 mmHg after 300-ml fluid loading, none of the 28 nonresponders after 300 ml responded to the additional 200-ml fluid loading.

## Discussion

The main finding of this large multicenter study of 65 shocked ARDS patients with neither arrhythmia nor spontaneous respiratory activity is that the performance of Δ_RESP_PP is poor in this clinical situation. Because fluid responsiveness prediction is of utmost importance in ARDS, we attempted unsuccessfully to improve Δ_RESP_PP performance by (1) its indexation, (2) analyzing different cutoffs for Δ_RESP_PP or fluid responsiveness definition or (3) identifying subgroups where Δ_RESP_PP may perform better.

Huang *et al.*'s study [17], including 22 patients, specifically addressed the issue of Δ_RESP_PP performance in ARDS and reported a similar AUC (0.77) for Δ_RESP_PP as in our population (0.75 (CI_95_: 0.62 to 0.085)). In our study, the AUC was not good, as the lower bound of the 95% confidence interval was below 0.75 [27]. Partly because confidence intervals for AUCs were not reported in Huang *et al.*'s study [17], it was considered that these authors' conclusion (that Δ_RESP_PP remains a reliable predictor of fluid responsiveness for ARDS patients ventilated with low Vt and high PEEP) was a misinterpretation [28,29]. In a large, multicenter population of ARDS patients, our results are similar to those of De Backer *et al. *[10], who found, in 33 patients (97% ARDS patients) receiving Vt <8 ml/kg, that Δ_RESP_PP did not perform better than PAOP. Other authors also observed this low performance of Δ_RESP_PP in case of low Vt. One can reasonably assume that many patients in those studies had ARDS, despite the lack of specific subgroup analysis [11,30]. Again, the complex pathophysiology of transmission of airway pressure changes to intrathoracic vascular structures [12,14,15] justified analyzing specifically the performance of Δ_RESP_PP in ARDS patients.

**Figure 5:**
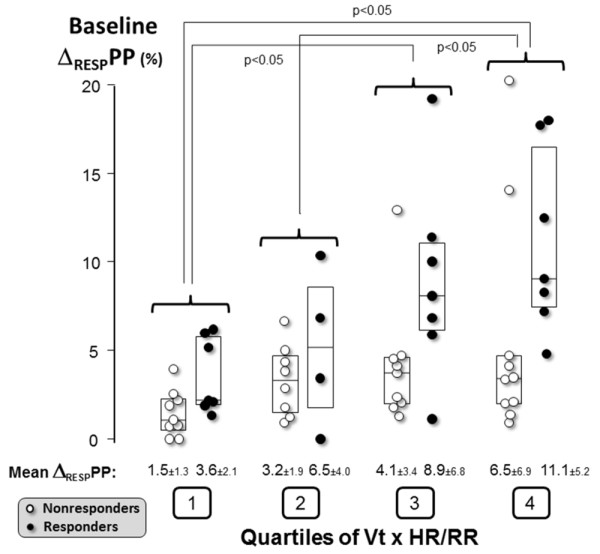
**Baseline Δ_RESP_PP according to Vt and HR:RR ratio**. Beyond chest wall compliance, Δ_RESP_PP is influenced by Vt [10], HR:RR ratio [16] and fluid responsiveness status. This is confirmed in our study population by using a composite index including these respiratory settings: Vt × HR:RR ratio. Two-way analysis of variance disclosed that the product of Vt × HR:RR ratio and the responder versus nonresponder status independently influenced the value of Δ_RESP_PP (*P *= 0.0013 and *P *= 0.0014, respectively). The results of post hoc tests (Fisher's procedure of least significant difference) between quartiles of (Vt × HR:RR ratio) are shown. With regard to the need for this physiological analysis, patients with ultrasonographic evidence of acute *cor pulmonale *(*n *= 4) were excluded. Vt, tidal volume; HR, heart rate. RR, respiratory rate; Δ_RESP_PP, respiratory changes in pulse pressure. Responders are defined as those patients with a 10% increase in cardiac output after 500-ml volume expansion. The central boxes represent the values from the lower to the upper quartile (25th to 75th percentile). The middle line represents the median value.

Interestingly, our mean Δ_RESP_PP was low at baseline (5.2%) compared with most studies exhibiting values close to 12% [2] (6% to 10% in ARDS patients [10,17]). Many causes can be identified to explain this low baseline Δ_RESP_PP value. First, it may be a consequence of including patients already resuscitated. Indeed, large volume expansion before inclusion (not recorded) may explain the low variations in blood pressure waveform we observed. However, despite this initial resuscitation, 40% of our patients were still fluid responders. Second, as previously shown [7,8,10,11], the low Δ_RESP_PP may also be related to the low Vt used in our population (6.9 ± 0.95 ml^-1 ^kg^-1^) compared with other studies reporting values of at least 8 ml^-1 ^kg^-1 ^[1,4,31-36]. Third, beyond their Vt dependency, breath-related indices also depend on the RR, and more specifically on the HR:RR ratio [16]. Again, our respiratory settings (RR, 24 ± 6/minute; HR:RR ratio, 4.5 ± 1.6) differed from those previously reported, with values ranging from 8 to 17/minute for mean RR and from 5 to 8 for mean HR:RR ratio [8,31-33,36]. It is noteworthy that these two limitations of Δ_RESP_PP (low Vt and high RR) often come together in particular in case of ARDS. Figure 5 illustrates the impact of Vt and HR:RR ratio on Δ_RESP_PP in our population.

Beyond these limitations (low Vt and high RR) causing false-negative cases of Δ_RESP_PP, false-positive cases may also arise because of a common phenomenon during ARDS: pulmonary artery hypertension [37,38] and/or right ventricular dysfunction [39]. We only searched for marked ultrasonographic signs of acute *cor pulmonale *(arrows in Figure 1). Performing more sophisticated measurements of right ventricular function (for example, peak systolic velocity of tricuspid annular motion) would have sensitized the detection of this restriction for Δ_RESP_PP usefulness [39]. It is noteworthy that pulmonary artery hypertension and/or right ventricular failure may be an even more frequent limitation of Δ_RESP_PP in case of later or more severe ARDS (PaO2/FiO2 <70) than patients whom we included.

Moreover, changes in chest wall compliance may also affect Δ_RESP_PP, positively or negatively. Decreased chest wall compliance, observed in cases of intraabdominal hypertension (extrapulmonary ARDS) [40] increases respiratory pleural pressure variations for a given Vt [14,15]. Thus, Δ_RESP_PP may be higher and present false-positive results in this situation. At the opposite, chest wall compliance may be increased through the use of muscle relaxants, which was the case in 40% of our patients, and then induce reduced intrathoracic pressure swings and therefore potential false-negative Δ_RESP_PP results. The lack of measurement of chest wall compliance in our patients (that is, no esophageal pressure measurement) precluded precise analysis of this factor. Nevertheless, using PAOP as a surrogate for esophageal pressure measurements, we performed some physiological analysis which allowed us to gain some insight into this issue.

Our findings do not confirm the hypothesis according to which, owing to ARDS-induced decrease in lung compliance, a small Vt (<8 ml/kg) may cause sufficient changes in intrathoracic pressure, allowing Δ_RESP_PP to perform well in this population [13]. Actually, ARDS-induced increase in lung stiffness is indeed associated with an increased airway driving pressure (by increased Pplat) for a given Vt [14], but the primary determinants of pleural pressure variations (and then of Δ_RESP_PP) have been shown to be the magnitude of Vt and chest wall compliance (both of them ruling the compression of the cardiovascular structures), regardless of lung compliance [14]. Indeed, using changes in PAOP as a surrogate for pleural pressure variations [41], we found that Δ_RESP_PP tended to perform markedly better in patients with high ΔPAOP (Figure 4A), illustrating the importance of high Vt and low chest wall compliance for Δ_RESP_PP to be useful. Indeed, in our analysis (with the limits of using ΔPAOP as a surrogate), respiratory changes in PAOP represent the ratio of Vt/chest wall compliance (detailed calculation in Additional file 1).

The rather good AUC (0.81 (CI_95_: 0.64 to 0.93)) that we found for Δ_RESP_PP/ΔPAOP (in the subset of Swan-Ganz catheter carriers) suggests that a more precise approach of pleural pressure swings may be a more interesting way to correct the crude Δ_RESP_PP and to improve its predictive ability. Not surprisingly, and as previously reported in case of low Vt [11], no improvement was observed in Δ_RESP_PP performance when it was corrected for airway driving pressure. Moreover, there was no marked evidence of better performance of Δ_RESP_PP in cases of high airway driving pressure (Figure 4B), reminding us that this parameter is not a major determinant of Δ_RESP_PP.

Our ARDS patients exhibited higher values of respiratory system static compliance (total of lung and chest wall compliance) than values usually reported in ARDS patients (40 versus 26 to 30 ml/cmH_2_O) [10,17,42]. There are three potential explanations for this difference: (1) because the PEEP level was not fixed by protocol, some patients may have had PEEP levels high enough to optimize recruitment and respiratory compliance [42]; (2) patients were studied at the early phase of ARDS (Table 1), and lung compliance is classically lower in late ARDS; and 3) we did not include the patients with the most severe cases of ARDS (PaO_2_:FiO_2 _ratio <70) for safety reasons. Of note, Δ_RESP_PP showed similar performance in patients with respiratory system static compliance below or above its median value (Additional file 1), preventing the use of this parameter to identify patients in whom Δ_RESP_PP might perform better. Because of higher respiratory system compliance, our airway driving pressure was in the lower reported range (13.7 versus 14 to 17 cmH_2_O) [10,17,42]. However, our mean Vt value was slightly higher (6.9 versus 6.3 to 6.4 ml/kg) [10,17,42]. Again, as Δ_RESP_PP is mostly influenced by the Vt rather than the airway driving pressure [7,10,14], one would have expected even better performance of Δ_RESP_PP than that reported in similar previous works.

In our population, the best cutoff value for Δ_RESP_PP was 5%, that is, close to that previously reported in ARDS patients with low Vt [10]. Another explanation for the poor ability of Δ_RESP_PP to predict fluid responsiveness may be that this low cutoff exposes it to errors in measurements because of low signal-to-noise ratio [12]. Of note, numerical recordings of Δ_RESP_PP in ARDS patients [10,17] did not lead to better performance than using high-resolution paper tracings, as we did.

For the same reasons developed for Δ_RESP_PP, we found that the other breath-related, blood pressure-derived indices, dDown and SPV, were of similar poor performance in predicting fluid responsiveness in our ARDS population. Before using fluid responsiveness prediction tools, one has to identify patients who may actually benefit from having their CO increased by fluids. In an overall population, many fluid responders actually do not need any fluids (that is, no need for an increase in CO). All of our patients were in acute circulatory failure and most presented signs of tissular hypoperfusion (oliguria in 34%, mottled skin in 34% and hyperlactatemia in 38%), suggesting that they may benefit from volume expansion, but baseline CVP (11 ± 4 mmHg) and PAOP (12 ± 4 mmHg) were unhelpful (Figure 2) [43]. It is precisely in these patients, that is, those with persistent circulatory failure despite initial resuscitation, that other indices are required; but Δ_RESP_PP is disappointing in patients with ARDS. In this situation, a fluid challenge may be performed [44]. Thus, during volume expansion, an increase in CVP ≥2 mmHg is considered to reflect that the Frank-Starling mechanism of the heart has been tested [43]. Interestingly, among the 40 patients who fulfilled this CVP change criterion after 300-ml volume expansion, none of the 28 nonresponder patients responded after 300 ml to the additional 200-ml volume expansion. Therefore, performing careful fluid challenges while monitoring both CVP and CO may be a safe way to limit undue fluid loading during ARDS.

## Conclusions

In our population of patients with early ARDS who were receiving protective mechanical ventilation, partly because of insufficient changes in pleural pressure, Δ_RESP_PP performed poorly in predicting fluid responsiveness. Fluid management in patients with ARDS may rely on fluid challenges.

## Key messages

• Respiratory variations of pulse pressure (Δ_RESP_PP) perform poorly in predicting fluid responsiveness in patients with ARDS.

• Both low tidal volume (by decreasing respiratory pleural pressure changes) and low HR:RR ratio downplay the performance of Δ_RESP_PP.

• Respiratory changes in pleural pressure, but not airway driving pressure, are the main determinant of Δ_RESP_PP.

• No simple means of improving Δ_RESP_PP performance was found.

• Because optimal fluid management is of utmost importance in ARDS patients, clinicians have to rely on other means, such as fluid challenges, for this purpose.

## Abbreviations

Δ_RESP_PP: respiratory variations in pulse pressure; ΔPAP: respiratory changes in pulmonary artery pressure; ΔPAOP: respiratory changes in pulmonary artery occlusion pressure; ARDS: acute respiratory distress syndrome; AUC: area under the receiver-operating characteristic curve; CO: cardiac output; CVP: central venous pressure; dDown: difference between the average, over three consecutive respiratory cycles, of the minimal value of systolic blood pressure during a respiratory cycle and the value of systolic blood pressure during apnea; HR: heart rate; LR+: positive likelihood ratio; LR: negative likelihood ratio; LSC: least significant change; PAOP: pulmonary artery occlusion pressure; PAOPtm: transmural pulmonary artery occlusion pressure; PEEP: positive end-expiratory pressure; Pplat: plateau pressure; RR: respiratory rate; SPV: respiratory changes in systolic arterial pressure over three consecutive respiratory cycles; Vt: tidal volume.

## Competing interests

The authors declare that they have no competing interests.

## Authors' contributions

KL, SE and TB contributed to the conception and design of the study. KL, SE, DBL, IR, EM, PFD, AL and TB contributed to the acquisition of data. KL, SE, MW, BR and TB contributed to the drafting and revision of the manuscript.

## Supplementary Material

Additional file 1 **Additional data and figures**. Impact of several clinical factors on the performance of Δ_RESP_PP: subgroup comparisons according to respiratory system compliance, norepinephrine dosage, neuromuscular blocking agent use and site of the artery catheter. Impact of the definition of fluid responsiveness on the performance of Δ_RESP_PP, individual values of baseline static and breath-derived indices in responders and nonresponders using the 15% cutoff for cardiac output to define fluid responsiveness, performance of Δ_RESP_PP using the 15% cutoff for cardiac output to define fluid responsiveness. Impact of chest wall compliance on Δ_RESP_PP provides additional comments to Figure 4. AUC, area under the receiver-operating characteristic curve; Δ_RESP_PP, respiratory changes in pulse pressure. Click here for file
