# Supplementary material for: Respiratory pulse pressure variation fails to predict fluid responsiveness in acute respiratory distress syndrome
Source: Crit Care. 2011 Mar 7;15(2):R85. doi: 10.1186/cc10083 (PMC3219343; doi:10.1186/cc10083)
Supplement: Additional file 1 — Additional data and figures. Impact of several clinical factors on the performance of ΔRESPPP: subgroup comparisons according to respiratory system compliance, norepinephrine dosage, neuromuscular blocking agent use and site of the artery catheter. Impact of the definition of fluid responsiveness on the performance of ΔRESPPP, individual values of baseline static and breath-derived indices in responders and nonresponders using the 15% cutoff for cardiac output to define fluid responsiveness, performance of ΔRESPPP using the 15% cutoff for cardiac output to define fluid responsiveness. Impact of chest wall compliance on ΔRESPPP provides additional comments to Figure 4. AUC, area under the receiver-operating characteristic curve; ΔRESPPP, respiratory changes in pulse pressure. [file cc10083-S1.DOC]

**ADDITIONAL DATA**

**Impact of several clinical factors on the performance of Δ_RESP_PP**

Δ_RESP_PP performed similarly (p>0.4) in the subgroups of patients:

- with a **respiratory system compliance** higher or lower than its median value (37.5 ml/cmH_2_O): AUC of 0.79 (0.61-0.91) and 0.71 (0.53-0.86) respectively.
- with **norepinephrine dosage** higher (n=27) or lower (n=38, including 12 patients not receiving any catecholamine) than its median value of 0.394 µg/kg/min: AUC of 0.82 (0.62-0.94) and 0.70 (0.53-0.83), respectively.
- receiving **neuromuscular blocking agents** (n=26) or not: AUC of 0.75 (0.54-0.90) and 0.75 (0.59-88), respectively.
- carrying a **radial** (n=14) or a **femoral** (n=51) **artery catheter**: AUC of 0.81 (0.52-0.96) and 0.72 (0.58-0.84), respectively.
- when excluding patients carrying either a pulmonary artery catheter with severe **tricuspid regurgitation** (n=2) or a PiCCO™ system with a severe **mitral and/or tricuspid and/or aortic regurgitation** (n=1) (per American Heart Association definition).

**Impact of the definition of fluid responsiveness on the performance of Δ_RESP_PP** Many studies in the field of fluid responsiveness prediction used a cardiac output (CO) cutoff of 15% to define fluid responders. We provided strong evidence in favor of the use of a 10% cutoff when using cold bolus thermodilution with inline temperature measurement based on least significant change analysis. Nevertheless to allow full comparison with other works performed in the field of fluid responsiveness we provide below full data using the traditionally used 15% cutoff (complement to table 3).

The performance of Δ_RESP_PP was poor when using the 15 % cutoff for volume expansion-induced change in cardiac output to define responsiveness and was of similar poor clinical value than when using a 10% cutoff (ADDITIONAL figure 1 and 2).

**ADDITIONAL Figure 1:** **Individual values of baseline static and breath-derived indices in responders and nonresponders (*15 % cutoff for cardiac output to define fluid responsiveness*)**

**
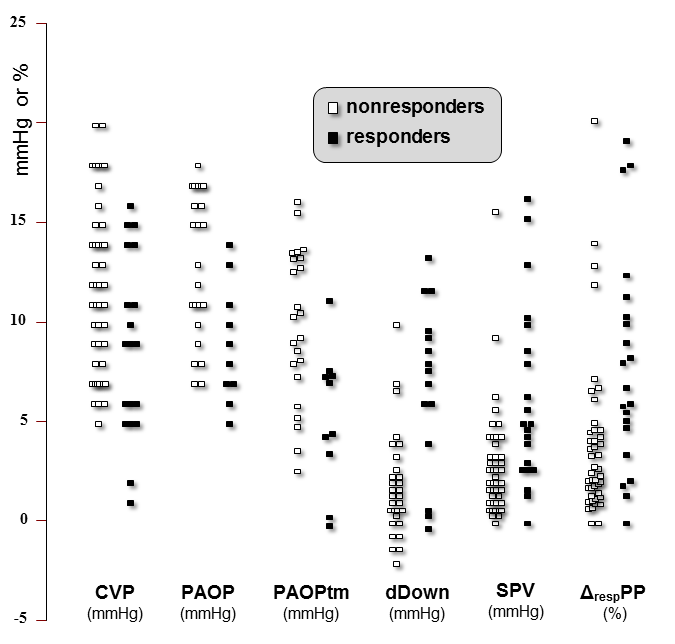
**

Legend: CVP: central venous pressure, PAOP: pulmonary artery occlusion pressure, PAOPtm: transmural pulmonary artery occlusion pressure (see text for details), Δ_RESP_PP: respiratory changes in arterial pulse pressure, dDown: expiratory decrease in systolic arterial pressure, SPV: respiratory changes in systolic arterial pressure.

**ADDITIONAL Figure 2:** **Performance of Δ_RESP_PP in shocked ARDS patients (n=65), *using the 15 % cutoff of cardiac output to define fluid responsiveness.***


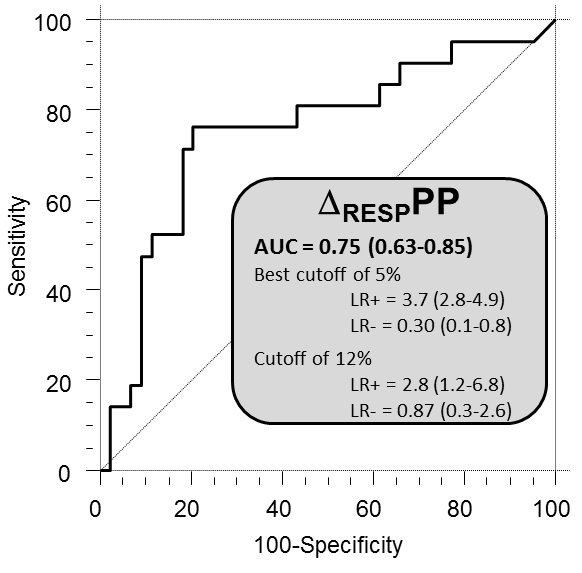


Legend: Receiver-operating characteristic (ROC) curve obtained for respiratory changes in pulse pressure (Δ_RESP_PP) to predict a *15 % increase in cardiac output* after 500 ml volume expansion. ARDS: acute respiratory distress syndrome. AUC: area under the ROC curve. LR+: positive likelihood ratio. LR-: negative likelihood ratio.

**Impact of chest wall compliance on Δ_RESP_PP**

In order to analyze more precisely the physiology of Δ_RESP_PP it would be of interest to evaluate the chest wall compliance as this is the actual determinant of Δ_RESP_PP alongside with the Vt. This requires estimation of respiratory changes in pleural pressure which are usually evaluated using an esophageal pressure measurement. As placing an esophageal balloon for this purpose was too cumbersome in this multicenter clinical study, respiratory changes in pleural pressure were estimated using respiratory changes in PAOP as a surrogate.

With these approximations, the value of the chest wall compliance is computed as follows:

Chest wall compliance = Vt / respiratory changes in PAOP.

Lung compliance may be calculated as follows: Lung compliance = Vt / (Driving pressure – respiratory changes in PAOP).

As Vt and chest wall compliance are the two major determinants of respiratory changes in right atrial pressure and thus in Δ_RESP_PP, one may combine those two factors and evaluate Δ_RESP_PP performance as a function of the ratio: Vt / chest wall compliance = respiratory changes in PAOP.

The results are presented in figure 4 of the main manuscript.
